# Double-blind, placebo-controlled pilot trial of L-Leucine-enriched amino-acid mixtures on body composition and physical performance in men and women aged 65–75 years

**DOI:** 10.1038/ejcn.2015.91

**Published:** 2015-06-17

**Authors:** T Ispoglou, H White, T Preston, S McElhone, J McKenna, K Hind

**Affiliations:** 1 Carnegie Faculty, School of Sport, Leeds Beckett University, Leeds, UK; 2 Scottish Universities Environmental Research Centre, University of Glasgow, Glasgow, Scotland

## Abstract

**Background/Objectives:**

Adequate protein intake is essential to retaining muscle and maintaining physical function, especially in the elderly, and L-Leucine has received attention as an essential amino acid (EAA) that enhances protein retention. The study's aim was to compare the efficacy of EAA mixtures on lean tissue mass (LTM) and functional performance (FP) in a healthy elderly population.

**Subjects/Methods:**

Thirty-six subjects (65–75 years) volunteered to receive capsules with EAAs (Groups A and B containing 20% and 40% L-Leucine, respectively) or placebo (lactose containing 0% L-Leucine, Group C) for 12 weeks. The daily amount ranged from 11 to 21 g (0.21 g/ kg/day) and was taken in two equal dosages alongside food, morning and evening. Main outcomes measured before and after intervention were LTM and FP (30-s arm-curl test; 30-s chair-stand test (30-CST); 6-min walk test (6-WT); and handgrip strength). Secondary outcomes included dietary intakes and physical activity.

**Results:**

Twenty-five subjects (11 male and 14 female) completed the study (Group A, *n*=8; Group B, *n*=8; Group C, *n*=9). Gains associated with medium effect sizes were noted in LTM (Group B, 1.1 ±1.1%, *P*=0.003) and FP (Group A in 30-CST (11.0±11.5%, *P*=0.02) and 6-WT (8.8±10.0%, *P*=0.02); Group B in 6-WT (5.8±6.6%, *P*=0.03) and a trend in 30-CST (13.2±16.0, *P*=0.06)). Significant differences between groups were not observed in secondary outcomes.

**Conclusions:**

Twice-daily supplementation of EAAs containing 20% or 40% L-Leucine improved aspects of functional status and at the higher level improved LTM. Further work to establish change in a larger sample and palatable supplemental format is now required.

## Introduction

Sarcopenia is a complex and multifactorial syndrome associated with significant clinical, social and economic consequences. With an ageing population, age-related sarcopenia has increased, and estimated prevalence rates are 13% in 60- to 70-year-old people and up to 50% in people aged 80 years and over.^1, 2^ Although there is no consensus for the definition of sarcopenia, the European Working Group on Sarcopenia in Older People (EWGSOP) has based the diagnostic criteria on the presence of low muscle mass, together with either low muscle strength or low physical performance.^3^ Maintaining muscle mass and physical function is fundamental to promoting health and independence with age. It has particular relevance for the prevention of falls, fracture and disability. However, the identification of effective treatments for age-related sarcopenia represents an ongoing challenge.

Some of the most promising interventions have been nutritionally based.^4, 5^ Adequate protein intake is considered essential, and reports suggest that the current recommended daily allowance of 0.8 g/ kg/day is insufficient for the general population.^6, 7^ Instead, prophylactic amounts of 1.0–1.6 g/ kg/d and a larger proportion of high-quality protein in a meal (25–30 g) rather than simply increasing general total protein intake are proposed to meet the increased requirements of older people and to prevent sarcopenia.^8, 9, 10^ Conversely, protein intakes decrease with age owing to changes in taste, difficulties in chewing, swallowing and age-related anorexia,^11, 12^ presenting practical challenges for achievement of higher intakes.

Research to determine the quantities of proteins and amino acids (AAs) required for older people with existing sarcopenia or those ‘at risk' is a priority,^3^ as is their formulation, palatability and delivery. The AA L-Leucine (leucine) has shown promise to date, and it is associated with enhanced postprandial protein retention.^13^ Leucine induces an upregulation of mRNA translation, an increase in muscle protein synthesis (PS) and accretion and it can reverse the age-related blunted response of muscle PS.^14, 15^ Studies in humans have shown that muscle PS in the elderly is superior when increasing the leucine content in a standard mixture of essential amino acids (EAAs).^16^ Taking also into account that with increasing age there is an uncoupling of the mechanism that regulates protein balance and consequently a reduced ability to use dietary protein for muscle synthesis,^17^ it is probable that this is mediated through a mechanism involving leucine.

Longitudinal nutrition intervention studies, exploring the effectiveness of essential EAAs, leucine and EAAs enriched with leucine, have presented conflicting findings.^18, 19, 20^ The daily ingestion of 15 g of a standard mixture of EAAs (20% leucine) over a period of 3 months improved lean body mass in older sedentary women^18^ in contrast to a daily intervention of 7.5 g, which had no effect on lean mass.^19^ However, neither intervention resulted in increases in strength. Although studies have demonstrated the potential for leucine as a pharmaco-nutritional prophylaxis against age-related sarcopenia,^21, 22, 23, 24, 25^ the optimal amount and composition remains unclear. Studies to determine this more precisely are of clinical and practical importance, especially where they account for factors such as reduced physical activity (PA), which are known to be associated with increased anabolic resistance that cannot be reversed by increased availability of AAs alone.^26, 27, 28^

The purpose of this study was to compare the efficacy of EAAs enriched with different amounts of leucine on muscle mass and physical performance in elderly men and women. We hypothesised that an increase in the proportion of leucine in a modified mixture of EAAs would be of greater benefit than a standard mixture of EAAs or a placebo.

## Materials and methods

### Study design

This is a double-blinded randomised controlled pilot trial of the effects of mixtures of EAAs in retired men and women. The study was approved by the University Research Ethics Committee. Signed informed consent was provided by all subjects before testing and intervention.

### Subjects/Population

Thirty-six men (*n*=36) and women aged 65 to 75 years volunteered to take part, and twenty-five (*n*=25) completed all pre- and post-functional and body composition tests. None of them smoked, all were living independently and were of good health. Of the initial number (*n*=36), those with lactose intolerance, vascular disease, hypertension, diabetes, cardiac abnormalities and the presence of metabolic disease or injury were excluded from participation (*n*=3); others withdrew from the study outlining either personal reasons or inability to take the supplements (*n*=6), and data from two subjects were not included in the final analysis because of non-compliance with the experimental protocol.

### Protocol

Subjects received one of the following daily:

- Standard EAA mixture (20% leucine)
- Modified EAA mixture (40% leucine)
- Isocaloric placebo (lactose).

The supplementation period was 3 months and it was in accordance with EWGSOP recommendations. Primary outcomes were total lean mass and physical performance. Measurements were taken at baseline and immediately post intervention. A standardised health screening and a pre-exercise screening questionnaire, blood pressure, resting heart rate and oxygen saturation levels were taken at each time point.

### Supplements and diet

#### Supplements

Subjects ingested the isocaloric supplements in indistinguishable gelatine clear capsules (Size 00, CapsulCN) with breakfast and dinner. As lean body mass was improved after daily ingestion of 15 g of EAAs in sedentary elderly women,^18^ we made a decision to use the equivalent relative amount (0.21 g /kg/day). Compliance was monitored by recording the number of capsules that were not taken. The EAAs (Arndale Ingredients, South Shields, UK) were packed into drums (Direct Food Ingredients Ltd, Macllesfield, UK) and placed in capsules using a capsule filler (CN-300, CapsulCN, Franklin Lakes, NJ, USA). The composition of the EAA mixtures can be seen in Table 1. A decision was made not to reduce the concentration of the branched chain AAs (BCAAs) isoleucine and valine in the enriched mixture; high intakes of leucine may increase the oxidation of isoleucine and valine and therefore limit their availability to synthesise new protein, and reduced isoleucine availability may decrease leucine transport into the cells.^29, 30, 31^ Therefore, we reduced only the percentage of the remaining EAAs in the modified mixture. The amount of ingested leucine remained below the upper tolerable and safe limit in humans of 0.53 g/kg/day,^32^ taking into account that the daily relative leucine intake in the enriched mixture was 0.08 g/kg/day. In absolute terms, a person weighing 80 kg would need to receive 16.8 g of EAAs on a daily basis, of which 6.72 g would be in the form of leucine.

#### Diet

In week 1 of supplementation, subjects who completed the intervention period (*n*=25) recorded all food and drinks consumed over a 3-day period. The process was repeated post intervention; however, 6 subjects failed to return their food diaries. Subjects received guidance on recording food and drink by household measures and weight, and subjects were asked to retain all packaging and food labels. Food diaries were coded and dietary analysis was undertaken using NetWISP version 3.0 (Tinuviel Software, Warrington, UK). Macronutrient and alcohol intakes were obtained, each expressed as the percentage of total energy intake. Total energy intake was also expressed as a percentage of estimated average requirements. Protein, iron, calcium and vitamin D were expressed as a percentage of Reference Nutrient Intake.^33^ Protein was also calculated in g/kg/day.

### Body composition

Subjects were measured wearing light-weight clothing with jewellery removed. Height was determined with a stadiometer (SECA, Birmingham, UK), and body weight was recorded by electronic scales (SECA). Whole-body composition was assessed using dual-energy X-ray absorptiometry scan (Lunar iDXA, GE Healthcare, Madison, WI, USA) with calibration checked daily using the GE Lunar calibration phantom. Precision for measurements in our unit are 0.5%, 0.8% and 0.6% for lean tissue, fat and bone mass, respectively.^34^

### Functional performance (FP) testing and PA levels

Tests were delivered in the following order: 30-s arm-curl test, 30-s chair-stand test (30-CST), handgrip strength test and 6-min walk test (6-WT).^35^

Techniques adopted have been described previously.^35, 36^ For familiarisation purposes, subjects performed sub-maximum efforts before maximal testing. Ratings of perceived exertion were recorded at the end of each exercise and attempt.^37^ Neoprene dumbbells (5 and 8 lbs for women and men, respectively) and a 44 -cm-height straight-back chair without arm rests were used during the 30-s arm-curl test and the 30-CST, respectively. A digital handheld dynamometer (Takei, Scientific Instruments Ltd, Niigata, Japan) recorded isometric strength of the dominant arm with values reported in the results section expressed relative to body weight (N/kg bw). During the 6-WT, subjects walked their maximal distance (50 m, 20 × 5 m^2^ rectangle) in 6 min.

A 7-day physical activity recall^38^ was used to estimate general PA levels.

### Blood sampling and analysis

A sub-group (*n*=5, *n*=4, and *n*=6 from groups A, B and C, respectively) volunteered for measurement of plasma concentrations of BCAAs and the non-essential AAs Alanine, Glycine and Proline. In an attempt to reduce attrition, blood sampling was not compulsory; therefore, blood was drawn from a convenience sample in each group. Subjects remained seated in a reclined position for 5 min before 2 ml of blood was drawn from one of the brachial, medial cubital or radial veins. Samples were drawn into 3.5- ml fluoride oxalate BD vacutainer tubes (Becton Dickinson and Company, Franklin Lakes, NJ, USA) and centrifuged for 10 min at 3000 revolutions/minute (ALC multispeed centrifuge PK 131R, ALC, Milan, Italy). Two aliquots of plasma were stored at −80 °C. Before analysis, each sample was thawed, an internal standard was added and proteins were removed by ultrafiltration. The ultrafiltrate was acidified and the sample was subjected to cation exchange clean-up, with elution in ammonium hydroxide. Samples were dried down and derivatised for gas chromatography mass spectrometry analysis. Plasma AAs were analysed by gas chromatography mass spectrometry after ultrafiltration, cation exchange and derivatisation as ethoxycarbonyl ethyl esters. Nor-leucine was used as an internal standard for quantification of the neutral AAs.

### Statistics

SPSS statistical software was used for analysis (PASW 18, SPSS, Hong Kong, China). Data are presented as means (±standard deviations). Normality was assessed using the Shapiro–Wilk Test, and data violating homogeneity assumptions were assessed via a Kruskal–Wallis test. One-way analysis of variance (ANOVA) tested for differences at baseline and compared mean percentage differences between groups A, B and C; significant interactions were explored using the Holm–Bonferonni *post-hoc* adjustment. Repeated-measures ANOVA was used test for body composition, FP, PA and RPE main effects; paired *t*-tests were used for within-group comparisons (baseline to post-intervention) when ANOVA resulted in significant main effects or significant trends. For non-parametric data (including RPE), the Wilcoxon signed rank test was used. Statistical comparisons for body composition, FP variables and RPE were also performed using Cohen's Effect Size with threshold values for small (0.2), medium (0.6), large (1.2), very large (2.0) and extremely large (4.0) effects. Statistical significance was inferred as *P*<0.05.

## Results

### Demographic data and body composition

The age and anthropometric characteristics are reported in Table 2 and the body composition variables in Table 3. Repeated-measures ANOVA revealed no time by participant interaction effects (F(2,22)=0.529, *P*=0.596); however, main time effects approached significance (F(1, 22)=3.643, *P*=0.069). Over the 3 months, lean tissue mass (LTM) in Group B (modified EAA (40% leucine)) increased significantly compared with baseline (*t*(7)=−2.695, *P*=0.031).

### FP, PA and ratings of perceived exertion (RPE)

FP and average RPE ratings are given in Table 4. Significant differences were not observed between groups in the mean percentage changes in scores for the 30-s arm-curl test (F(2,22)=1.776, *P*=0.193), the 30-CST (F(2,22)=0.781, *P*=0.470), the handgrip strength test (F(2,22)=1.271, *P*=0.300) and the 6-WT (F(2,22)=2.225, *P*=0.132). In contrast, mean percentage reductions in RPE were significant (F(2,22)=4.683, *P*=0.020); Group C (Placebo) had the greatest reductions in RPE, which was significantly different from Group A (*P*=0.023).

The EAA groups demonstrated significant improvements in FP compared with baseline. In Group A (standard EAA (20% leucine)), improvements were observed in the 30-CST (*t*(7)=−3.211, *P*=0.015)) and 6-WT (*t*(7)=−2.859, *P*=0.024)); in Group B (modified EAA (40% leucine)), in the 6-WT (*t*(7)=−2.659, *P*=0.032) and in the 30-CST, improvements almost reached statistical significance (*t*(7)=−2.173, *P*=0.066). Baseline differences between Groups A (standard EAA (20% leucine)) and C (placebo) in the 30-CST (F(2,22)=6.3, *P*=0.007) remained significant at the end of the intervention (F(2,22)=4.1, *P*=0.03). Mean improvements in FP tests in the EAA groups were also associated with medium to large effect sizes (Table 4). No additional effect size was noted for higher-dose leucine supplementation (Group B) compared with standard dose (Group A) in any of the FP tests (Table 4). RPE was reduced in Group C (placebo) by the end of the intervention (*Z*=−2.668, *P*=0.008).

PA at baseline was similar across all groups, with the total duration spent in activities being 7.4(±7.4), 8.97(±6.85) and 7.14(±3.71) h for the A, B and C groups, respectively. Towards the end of the intervention, Groups A, B and C increased on average their total PA levels by 104.7(±344.3)%, 30.6(±107.2)% and 114.3(±128.4)%, respectively. Mean percentage changes were not significantly different between groups. However, Group C (Placebo) increased total PA levels when compared with baseline (*t*(8)=−2.361, *P*=0.046).

### Nutrition

Percentage capsule intake was 75.0(±13.2)%, 75.8(±10.4)% and 85.8(±15.0)% for Groups A, B and C, respectively; however, it was not significantly different between groups (*P*=0.186). Energy intakes were sub-optimal, although no significant differences existed between groups (Table 5). Dietary protein intake (not including EAA supplementation) ranged between 0.95 and1.10 and 1.02–1.08 g/kg/day at baseline and post-intervention, respectively. The only difference observed was that Group C (placebo) had greater % carbohydrate intake at baseline (46.9%) than both EAA groups (Group A, 36.9% Group B, 41.7%), and the lowest alcohol intake, although not significantly different from the EAA groups.

### Blood data

Because of non-compliance and drop-out, *n*=3 (Group A), *n*=4 (Group B) and *n*=6 (Group C) completed blood sampling. Plasma AAs at baseline (Table 6) were similar, with the exception of isoleucine and valine, which were significantly higher in Group B (modified EAA (40% leucine)) than in Group C (placebo). Moreover, isoleucine concentration in Group B was significantly higher than in Group A (standard EAA (20% leucine)). There was a reduction in the concentration of all AAs in Group B (modified EAA (40% leucine)), no significant changes in Group A and only glycine was significantly reduced in Group C (placebo). There were also significant differences in the changes from pre- to post- supplementation in isoleucine and valine. *Post-hoc* tests showed that reductions in both AAs in Group B (modified EAA (40% leucine)) were significantly different from the respective gains in Groups A and C. No significant differences were observed between Groups A and C.

## Discussion

As the first to investigate the comparative impact of two different doses of leucine enriched amino-acid mixtures in healthy older individuals, we report that the addition of standard (20%) and high level (40%) leucine supplements appears to significantly improve aspects of functional status, and at the higher level of 40% leucine, increases LTM.

Long-term supplementation studies examining the effectiveness of leucine and EAAs enriched with leucine for prevention of either age-related sarcopenia^18, 19^ or enhancement of LTM^20^ are few and remain equivocal. High-dose long-term leucine supplementation alone taken as 7.5 g of free leucine rather than as part of a mixed amino-acid mixture or meal has shown no benefit over placebo, with both groups increasing LTM by ~0.4 kg.^19^ This contrasts with the use of leucine-enriched AAs^16, 18, 22^ or the use of a whey protein, leucine-enriched supplement, which has demonstrated beneficial acute effects on muscle PS, and particularly at higher concentrations of 40%. Similarly, animal studies have also revealed that the amount of leucine required to maintain postprandial stimulation of PS may be higher in older animals.^23^ In our study, PS was not measured; however, we can speculate that the significant increase in LTM of subjects in Group B was probably because of enhanced rates of PS as a result of an increased leucine dose (leucine intake ~6 g/day) and in the presence of other EAAs.

The increase of 1.1% in LTM (Leucine 40%) was lower than a 3.9% improvement achieved with an 18.6% leucine-enriched amino-acid mixture administered to elderly sedentary women.^18^ Interestingly, Dillon *et al.*^18^ took no account of protein intakes at baseline, with the potential that this was lower than our study, with resultant greater gains in LTM at lower leucine concentrations. Our subjects also received EAA amounts relative to their body weight and not absolute amounts, which potentially may be more beneficial for subjects with low body weight. Moreover, and despite the fact that subjects in the study by Dillon *et al.*^18^ were instructed to refrain from making any drastic changes to their habitual PA levels, it is not guaranteed that this was the case; subjects in our study were given similar instructions, but subjects in the placebo group significantly increased their PA levels compared with baseline. Dillon *et al.*^18^ on the other hand did not report PA level data to confirm or refute their assumption that subjects maintained their normal activity levels throughout the 3-month supplementation period. Bearing also in mind that subjects in our EAAs groups managed only ~75% of the prescribed dosage, it is highly probable that LTM gains were sub-optimum when compared with the previous study.^18^ PA and habitual protein intake may contribute to a blunted muscle synthesis response to leucine supplementation.^28, 39^ When total dietary protein and leucine intakes are optimal, this may already be sufficient to maximise muscle protein synthetic response; therefore, additional supplementation may not be of any benefit.^19^ It is possible that in the context of our subjects' relatively high baseline protein intakes, only high-dose leucine^23^ or high-quality protein^40^ would positively have an impact on LTM. Certainly, our subjects were well nourished. Dietary protein (%RNI) exceeded that of UK and European adult population recommendation^41, 42^ and at intakes of 1–1.1 g/kg/day it just met the proposed higher intakes of 1–1.5 g/kg/day for the prevention of sarcopenia,^8^ implying that those in the EAA groups would hypothetically require the very highest upper level of 1.5 g/kg/day dietary protein in order to achieve benefit. Age-related issues associated with consumption of food^11, 12^ render achievement of this high protein intake unlikely. Even if this were achieved, the provision of extra protein may diminish the impact because of the potential of protein to enhance satiety and subsequently reduce energy intake.^43^ Although the standard EAA Group (20% leucine) received as much protein and EAAs as those receiving the modified EAA (40% leucine) supplement, the changes in LTM were trivial, suggesting that the percentage concentration of AAs in the 20% supplement was not optimised for muscle growth. Surprisingly, in the placebo group a small (0.8%) but non-significant increase in LTM was not accompanied by significant gains in FP. The potential reason for this discrepancy may be explained by the placebo group significantly increasing their PA levels, which was also associated with reduced RPE levels, compared with baseline, and in so doing, stimulated the known anabolic role of exercise^44^ and increased the utilisation of AAs for muscle PS through increased blood flow.^45^ Compliance to intervention is a major challenge in research of this nature, as demonstrated by our study. Despite the fact that LTM gains were lower than previously reported^18^ for a standard mixture of EAAs, our data showed a significant trend for greater gains in LTM when subjects were supplemented with a modified mixture enriched with leucine, which was not the case for a standard mixture and a placebo. Our study also highlighted that PA in particular is a confounding factor that can contribute to LTM gains. Therefore, it is recommended that future studies include regular monitoring and encouragement of trial conditions during the intervention duration. More specifically, there is a need for incorporating direct measures of PA levels such as accelerometers^46, 47^ rather than just relying on self-reported tools such as the 7-day recall, which may reasonably assess total and very hard PA levels in men; however, it is less accurate in women and when the majority of activities are light or moderate.^48^

The presentation of leucine appears to have a key role in its action. Increasing age results in an uncoupling of the regulatory mechanism for protein balance and a reduced ability to use dietary protein.^17^ In younger populations, a standard mixture of EAAs was shown to be as effective in stimulating muscle PS as an EAA mixture enriched with leucine (35%), although the higher leucine concentration also decreased muscle protein breakdown rate.^31^ A higher arterial leucine concentration did not result in increased leucine transport or intracellular availability, as the decrease in isoleucine may have been the limiting factor for leucine transport in the cell. In the majority of studies in which a mixture of EAAs was enriched with leucine, the concentration of the other BCAAs was reduced, potentially rendering the EAA mixture sub-optimal. In contrast, we maintained the relative proportion of isoleucine and valine as in a standard mixture, and administered alongside food. Our data suggest that increased dosage of leucine, BCAAs and EAAs through supplementation and food can benefit the functional status and body composition of elderly men and women. It is noteworthy that % alcohol intake was two or three times greater in the EAA groups than in the placebo. Alcohol has been shown to inhibit the ability of leucine to increase PS^49, 50^ and may have blunted the effects of leucine on accrual of LTM. Assuming that alcohol intake was similar in all groups, we would expect to observe greater gains in LTM of the EAA groups compared with the placebo. Concentrations of isoleucine and valine were significantly decreased at the end of the intervention, which is in agreement with other studies,^51, 52, 53^ although we cannot confirm whether this change was because of decreased rates in protein breakdown or increase in PS rates. In rats fed a BCAA mixture enriched with leucine (31%), a greater life span and increased mitochondrial synthesis in cardiac and skeletal muscle have been noted.^23^ This supports an additional benefit of leucine and/or EAA supplementation other than LTM gains alone. Certainly, there were significant performance gains in Group A (standard EAA (20% leucine)) that may potentially be influenced by changes at the mitochondrial level and is worthy of further investigation.

In contrast to other studies, subjects in the EAA groups improved FP, suggesting that supplementation with EAAs is an effective nutritional therapy for age-related sarcopenia with or without the presence of exercise,^54^ which is a known enhancer of effect. Studies of similar duration^18, 19^ indicate no impact of EAAs or leucine supplementation on strength. This is of no surprise, as subjects did not take part in a resistance exercise training intervention and the strength tests assessed one-repetition maximum strength rather than FP. In terms of bone health, our findings also suggest that supplementation with EAAs may improve bone mineral density, as there was an increase in bone mineral density, which was supported by medium effect sizes (0.5) for both the 20 and 40% groups, whereas this was not the case for the placebo group. Three months is a short duration over which to assess change in bone density owing to bone remodelling cycle dynamics; therefore, research over 6 months is required to further explore this potential.

Although the findings above lend support to continued use of our own leucine-enriched formulation in the longer term, there is a need for further research with larger sample sizes before concrete recommendations are made. Moreover, adherence rates of 74–83% suggest that supplement presentation and palatability require further evaluation and development. The high attrition rates (30%) and the reasons subjects outlined for their withdrawal, also lend further support to the need for further development of nutritional products that deliver EAAs in a more compact, palatable and acceptable way to an elderly population.

## Conclusion and recommendations

Our data support the potential prophylactic role of EAAs and in particular leucine and BCAAs for the treatment of sarcopenia. Twice-daily supplementation with 0.21 g/kg/day EAAs (modified EAA (leucine 40%)) alongside a diet providing adequate protein and energy resulted in significant gains in FP and LTM, which was not the case for the placebo. On the other hand, the standard mixture of EAAs (leucine 20%) was associated with FP gains in the absence of changes in LTM. Our findings support the higher protein recommendations proposed for older individuals. Further studies examining the impact of higher dietary protein intakes on diagnostic criteria of sarcopenia such as physical performance and LTM are required, as well as studies on how palatability and satiety might also be addressed.

## Figures and Tables

**Table 1:** Composition of essential amino-acid mixtures per 100 g

*Amino acids*	*Standard essential amino-acid mixture containing 20% leucine*	*Modified essential amino-acid mixture containing 40% leucine*
Histidine	10	5
Isoleucine	11	11
Leucine	20	40
Lysine	15	12
Methionine	3	2
Phenylalanine	15	7
Threonine	14	11
Valine	12	12

**Table 2:** Age and anthropometric characteristics of the subjects^a^

*Group*	*Age (years)*	*Height (m)*	*Weight (kg)*		*Body mass index (kg/m*^*2*^)	
			*Baseline*	*12 Weeks*	*Baseline*	*12 Weeks*
A *n*=8 (3 M, 5 F)	71.1±2.7	1.68±0.11	75.6±15.3	76.7±15.9	26.6±3.8	26.8±3.9
B *n*=8 (4 M, 4 F)	71.9±3.0	1.67±0.08	74.4±12.7	74.8±12.2	26.7±3.4	26.8±3.3
C *n*=9 (4 M, 5 F)	71.8±2.7	1.64±0.10	70.5±12.4	70.8±12.1	26.3±3.9	26.4±3.9
Average	71.6±2.7	1.66±0.09	73.4±13.1	74.0±13.1	26.5±3.6	26.7±3.6
F (2, 22)	0.169	0.425	0.332	0.426	0.024	0.166
*P*-value	0.846	0.659	0.721	0.658	0.976	0.848

Abbreviations: A, standard essential amino-acid mixture (containing 20% leucine); B, modified amino-acid mixture (containing 40% leucine); C, placebo; F, female; M, male.

a All values are means±standard deviation.

**Table 3:** Assessment of body composition at baseline and at the end of the intervention period^a^

*Body composition variables*	*Group A (*n=*8)*				*Group B (*n=*8)*				*Group C (*n=*9)*		
	*Baseline*	*Week 12*	*%*	*ES*	*Baseline*	*Week 12*	*%*	*ES*	*Baseline*	*Week 12*	*%*
*Total LTM (kg)*	44.8±7.1	44.8±7.2	0.2±2.4	−0.4	45.5±8.9	46.0±9.1^b^	1.1±1.1	0.2	42.6±8.9	43.0±9.3	0.8±1.3
*Total FM (kg)*	27.8±11.4	28.5±11.2	4.3±6.7	0.4	25.6±6.2	25.7±6.0	0.5±2.6	−0.2	24.9±9.9	25.1±9.7	1.5±6.3
*Percentage BF (%)*	36.0±10.5	36.7±9.7	3.3±6.1	0.5	34.8±6.6	34.7±6.5	−0.4±2.1	−0.2	35.2±11.2	35.2±11.0	0.4±4.7
*Total BMC (kg)*	2.6±0.5	2.6±0.5	0.1±1.4	−0.3	2.5±0.6	2.5±0.6	0.0±1.0	−0.4	2.4±0.5	2.4±0.5	0.4±1.0
*Total BMD (g/cm*^*2*^)	1.1±0.1	1.1±0.1	0.2±1.2	0.5	1.1±0.2	1.1±0.2	0.3±1.6	0.5	1.1±0.1	1.1±0.1	−0.4±1.1

Abbreviations: A, standard essential amino-acid mixture (containing 20% leucine); B, modified amino-acid mixture (containing 40% leucine); C, placebo; BMC, bone mineral content; BMD, bone mineral density; BF, body fat; ES, Effect Size; FM, fat mass; LTM, bone mineral-free lean tissue mass; %, mean percentage change from baseline to week 12.

a All values are means±standard deviations.

b Denotes significantly different from baseline value (*P*<0.05). ME=mean of the experimental group, MP=mean of the placebo group. ES Cohen's *d*=(ME-MP)/SD pooled).

**Table 4:** Assessment of functional performance and ratings of perceived exertion at baseline and at the end of the intervention period^a^

*Functional performance variables*	*Group A (*n=*8)*				*Group B (*n=*8)*				*Group C (n=9)*		
	*Baseline*	*Week 12*	*%*	*ES*	*Baseline*	*Week 12*	*%*	*ES*	*Baseline*	*Week 12*	*%*
30-ACT (no)	14.6±4.7	16.3±4.0	15.0±20.0	0.8	17.4±4.0	18.6±3.6	8.3±13.7	0.6	18.6±3.0	18.7±3.0	0.9±11.2
30-CST (no)	11.6±3.9^b^	12.8±3.9b,^c^	11.0±11.5	0.5	14.1±2.0	15.9±3.2	13.2±16.0	0.5	16.4±2.1	17.1±2.3	4.7±15.7
Relative HST (N/(kg.bw))	2.8±0.9	3.0±1.0	11.5±23.9	0.6	3.8±1.2	3.9±1.3	4.8±7.5	0.6	4.0±1.4	3.9±1.3	−0.2±8.9
6-WT (m)	501.6±101.1	539.0±83.0^c^	8.8±10.0	0.9	533.9±81.0	562.1±72.4^c^	5.8±6.6	0.7	586.0±85.0	593.0±82.1	1.4±4.5
Average RPE	11.3±1.7	11.0±1.5	−2.0±9.0^b^	1.3	11.7±1.0	11.1±1.6	−5.2±10.1	1.0	13.1±1.6	11.3±1.6^c^	−13.7±5.1

Abbreviations: A, standard essential amino-acid mixture (EAAs); B, EAAs containing 40% leucine; C, placebo; ES, Effect Size; HST, handgrip strength test; no, number of repetitions; RPE, rating of perceived exertion; %, mean percentage change from baseline to week 12; 30-CST, 30-s chair-stand test; 30-ACT, 30-s arm-curl test; 6-WT, 6-min walk test.

a All values are means±standard deviations.

b Denotes significantly different from placebo (*P*<0.05) ME= mean of the experimental group, MP=mean of the placebo group. ES Cohen's *d =(ME-MP)/SD pooled)*.

c Denotes significantly different from baseline value (*P*<0.05).

**Table 5:** Nutrient intakes at baseline and at the end of intervention

*Nutritional variables*	*Group A (*n=*5)*			*Group B (*n=*8)*			*Group C (*n=*6)*			*Pre- to post- supplementation difference across groups (ANOVA)*
	*Baseline*	*Week 12*	*Mean difference*	*Baseline*	*Week 12*	*Mean difference*	*Baseline*	*Week 12*	*Mean difference*	P
Energy (kcal)	1872±754	1811± 740	−61± 325	1787± 305	1754±363	−33.0±175	1845±411	1812±372	−33.4±325	0.96
Energy (Kcal) % EAR	97.4±35.3	94.8±33.0	−2.6±18.1	87.5±13.0	85.7±12.5	−1.8± 8.3	89.0±24.0	88.1± 20.8	−0.9±17.7	0.77
Total % fat	34.6±12.3	28.2± 8.6	−6.4±7.3	32.7±5.0	31.5±2.3	−1.2±5.8	31.5±5.2	32.3±5.3	0.8±6.5	0.98
Total % CHO	36.9±6.9^*^	43.1± 3.1	6.2±6.8^*^	41.7±3.0	42.2±8.2	0.5±2.7	46.9±6.0	47.8±5.4	0.9±3.3	0.05
Total % protein	14.1± 2.7	16.5±4.7	2.4±4.5	17.4±3.8	18.2±3.9	0.8±5.5	16.9±2.4	16.1±3.1	−0.8.± 2.6	0.34
Total % alcohol	14.3±17.4	12.1± 13.6	−1.8±4.1	8.2±6.1	8.2±4.5	0±4.7	4.7± 3.6	3.8±4.3	−0.9±2.9	0.21
Protein (g)	62.5±17.4	71.2±30.2	8.7±15.0	77.5±18.4	72.3±15.9	−5.2±16.7	77.8±19.2	78.6± 20.2	0.8±30.7	0.54
Protein(% RNI)	133.2±37.9	152.2±65.3	19.0±32.2	166±40.4	167±44.1	1.8± 65.7	165.6±39.1	150.1± 40.8	−15.6± 39.8	0.57
Calcium (% RNI)	121± 65.4	108±65.3	−13.0±28.6	109±42.4	107.7±31.7	−1.3±44.3	130±42.0	121± 41.1	−9.± 23.0	0.80
Iron (% RNI)	123.6±65.0	123.2±103.7	0.4±73.2	98.6±1.2	96.3±35.0	−2.3.± 43.0	116±34.5	110±43.7	−6.0±28.6	0.97
Vitamin D (% RNI)	20.6±19.4	39.8±64.0	19.2±68.2	13.7±5.5	46±51.2	32.3±50.8	34.5±14.0	29.5±19.5	−5.± 29.7	0.34

Abbreviations: ANOVA, analysis of variance; A, standard essential amino-acid mixture (containing 20% leucine); B, modified amino-acid mixture (containing 40% leucine); C, placebo; EAR, estimated average requirement; RNI, reference nutrient intake. All values are means±standard deviation.

Intakes were calculated according to the following UK reference values

EAR Energy=2330 kcal/day ( male 65–74 years), 2100 kcal/day (male 75+ years), 1900 kcal/day (female 65–74 years) and 1810 kcal/day (female 75+ years); RNI protein=53.3 g/day (male 50+ years) and 46.5 g/day (female 50+ years); calcium=700 mg/day (male and female 50+ years); iron=8.7 mg/day (male and female 50+ years) and vitamin D=10 μg/day (male and female 50+ years).

Denotes significantly different from placebo (*P<0.05).

**Table 6:** Concentration of amino acids (μMol/l) at baseline and at the end of intervention

*Amino acids*	*Group A (*n=*3)*			*Group B (*n=*4)*			*Group C (*n=*6)*			*Pre to post supplementation difference across groups (ANOVA)*
	*Baseline*	*Week 12*	*Mean difference*	*Baseline*	*Week 12*	*Mean difference*	*Baseline*	*Week 12*	*Mean difference*	P
*Leucine*	147.3±8.1	175.3±24.6	28.0±29.1	224.0±82.5	187.0±21.5	−37.0±75.3	142.5±36.4	145.8±23.0	3.3±33.3	0.250
*Isoleucine*	64.7±7.6	78.7±6.0	14.0±14.0	101.7±24.0a,^b^	65.3±9.6^c^	−36.5±15.4	61.7±10.8	66.2±11.4	4.5±14.7	**0.002**
*Valine*	192.0±23.6	219.7±43.7	27.7±46.1	259.5±41.5^a^	203.8±39.7^c^	−55.8±27.6	175.8±42.1	198.5±37.0	22.7±39.3	**0.017**
*Alanine*	223.0±93.2	250.7±84.8	27.7±84.2	254.5±47.7	212.3±54.4^c^	−42.3±23.0	241.3±46.1	259.7±39.1	18.3±33.8	0.119
*Glycine*	180.0±51.6	172.7±110.0	−7.3±59.1	186.0±31.4	156.3±17.9^c^	−29.8±18.5	193.2±73.1	223.5±96.7^c^	30.3±28.1	0.059
*Proline*	141.0±33.9	140.3±5.8	−0.7±39.0	132.5±12.7	114.8±22.9	−17.8±26.0	112.5±26.8	128.0±40.0	15.5±32.2	0.310

All values are means±standard deviation.

Abbreviations: ANOVA, analysis of variance; A, standard essential amino-acid mixture (EAAs); B, EAAs containing 40% leucine; C, placebo.

a Denotes significantly different from placebo (*P*<0.05).

b Denotes significantly different from group A (*P*<0.05)

c Denotes significantly different from baseline value (*P*<0.05).

## References

[bib1] 1Morley JE. Sarcopenia: diagnosis and treatment. J Nutr Health Aging 2008; 12: 452–456. 18615226 10.1007/BF02982705 PMC12892578

[bib2] 2von Haehling S, Morley JE, Anker SD. An overview of sarcopenia: facts and numbers on prevalence and clinical impact. J Cachexia Sarcopenia Muscle 2010; 1: 129–133. 21475695 10.1007/s13539-010-0014-2 PMC3060646

[bib3] 3Cruz-Jentoft AJ, Landi F, Topinkova E, Michel JP. Understanding sarcopenia as a geriatric syndrome. Curr Opin Clin Nutr Metab Care 2010; 13: 1–7. 19915458 10.1097/MCO.0b013e328333c1c1

[bib4] 4Tieland M, van de Rest O, Dirks ML, van der Zwaluw N, Mensink M, van Loon LJ et al. Protein supplementation improves physical performance in frail elderly people: a randomized, double-blind, placebo-controlled trial. J Am Med Dir Assoc. 2012; 13: 720–726. 22889730 10.1016/j.jamda.2012.07.005

[bib5] 5Arnarson A, Gudny Geirsdottir O, Ramel A, Briem K, Jonsson PV, Thorsdottir I. Effects of whey proteins and carbohydrates on the efficacy of resistance training in elderly people: double blind, randomised controlled trial. Eur J Clin Nutr 2013; 67: 821–826. 23486511 10.1038/ejcn.2013.40

[bib6] 6Wolfe RR, Miller SL. The recommended dietary allowance of protein: a misunderstood concept. JAMA 2008; 299: 2891–2893. 18577734 10.1001/jama.299.24.2891

[bib7] 7Houston DK, Nicklas BJ, Ding J, Harris TB, Tylavsky FA, Newman AB et al. Dietary protein intake is associated with lean mass change in older, community-dwelling adults: the Health, Aging, and Body Composition (Health ABC) Study. Am J Clin Nutr 2008; 87: 150–155. 18175749 10.1093/ajcn/87.1.150

[bib8] 8Morley JE, Argiles JM, Evans WJ, Bhasin S, Cella D, Deutz NE et al. Nutritional recommendations for the management of sarcopenia. J Am Med Dir Assoc 2010; 11: 391–396. 20627179 10.1016/j.jamda.2010.04.014 PMC4623318

[bib9] 9Volpi E, Campbell WW, Dwyer JT, Johnson MA, Jensen GL, Morley JE et al. Is the optimal level of protein intake for older adults greater than the recommended dietary allowance? J Gerontol A Biol Sci Med Sci 2013; 68: 677–681. 23183903 10.1093/gerona/gls229 PMC3660117

[bib10] 10Paddon-Jones D, Rasmussen BB. Dietary protein recommendations and the prevention of sarcopenia. Curr Opin Clin Nutr Metab Care 2009; 12: 86–90. 19057193 10.1097/MCO.0b013e32831cef8b PMC2760315

[bib11] 11Wakimoto P, Block G. Dietary intake, dietary patterns, and changes with age: an epidemiological perspective. J Gerontol A Biol Sci Med Sci 2001; 56 Spec No 2: 65–80. 11730239 10.1093/gerona/56.suppl_2.65

[bib12] 12Morley JE. Anorexia of aging: physiologic and pathologic. Am J Clin Nutr. 1997; 66: 760–773. 9322549 10.1093/ajcn/66.4.760

[bib13] 13Gryson C, Walrand S, Giraudet C, Rousset P, Migne C, Bonhomme C et al. “Fast proteins” with a unique essential amino acid content as an optimal nutrition in the elderly: growing evidence. Clin Nutr 2014; 33: 642–648. 24090685 10.1016/j.clnu.2013.09.004

[bib14] 14Koopman R, Wagenmakers AJ, Manders RJ, Zorenc AH, Senden JM, Gorselink M et al. Combined ingestion of protein and free leucine with carbohydrate increases postexercise muscle protein synthesis *in vivo* in male subjects. Am J Physiol Endocrinol Metab 2005; 288: E645–E653. 15562251 10.1152/ajpendo.00413.2004

[bib15] 15Leenders M, van Loon LJC. Leucine as a pharmaconutrient to prevent and treat sarcopenia and type 2 diabetes. Nutr Rev 2011; 69: 675–689. 22029833 10.1111/j.1753-4887.2011.00443.x

[bib16] 16Katsanos CS, Kobayashi H, Sheffield-Moore M, Aarsland A, Wolfe RR. A high proportion of leucine is required for optimal stimulation of the rate of muscle protein synthesis by essential amino acids in the elderly. Am J Physiol Endocrinol Metab 2006; 291: E381–E387. 16507602 10.1152/ajpendo.00488.2005

[bib17] 17Cuthbertson D, Smith K, Babraj J, Leese G, Waddell T, Atherton P et al. Anabolic signaling deficits underlie amino acid resistance of wasting, aging muscle. FASEB J 2005; 19: 422–424. 15596483 10.1096/fj.04-2640fje

[bib18] 18Dillon EL, Sheffield-Moore M, Paddon-Jones D, Gilkison C, Sanford AP, Casperson SL et al. Amino acid supplementation increases lean body mass, basal muscle protein synthesis, and insulin-like growth factor-I expression in older women. J Clin Endocrinol Metab 2009; 94: 1630–1637. 19208731 10.1210/jc.2008-1564 PMC2684480

[bib19] 19Verhoeven S, Vanschoonbeek K, Verdijk LB, Koopman R, Wodzig WK, Dendale P et al. Long-term leucine supplementation does not increase muscle mass or strength in healthy elderly men. Am J Clin Nutr 2009; 89: 1468–1475. 19321567 10.3945/ajcn.2008.26668

[bib20] 20Ispoglou T, King RFGJ, Polman RCJ, Zanker C. Daily L-Leucine supplementation in novice trainees during a 12-week weight training program. Int J Sport Physiol 2011; 6: 38–50. 10.1123/ijspp.6.1.38 21487148

[bib21] 21Rieu I, Sornet C, Bayle G, Prugnaud J, Pouyet C, Balage M et al. Leucine-supplemented meal feeding for ten days beneficially affects postprandial muscle protein synthesis in old rats. J Nutr 2003; 133: 1198–1205. 12672943 10.1093/jn/133.4.1198

[bib22] 22Rieu I, Balage M, Sornet C, Giraudet C, Pujos E, Grizard J et al. Leucine supplementation improves muscle protein synthesis in elderly men independently of hyperaminoacidaemia. J Physiol 2006; 575: 305–315. 16777941 10.1113/jphysiol.2006.110742 PMC1819434

[bib23] 23Dardevet D, Sornet C, Balage M, Grizard J. Stimulation of *in vitro* rat muscle protein synthesis by leucine decreases with age. J Nutr 2000; 130: 2630–2635. 11053498 10.1093/jn/130.11.2630

[bib24] 24Guillet C, Zangarelli A, Mishellany A, Rousset P, Sornet C, Dardevet D et al. Mitochondrial and sarcoplasmic proteins, but not myosin heavy chain, are sensitive to leucine supplementation in old rat skeletal muscle. Exp Gerontol 2004; 39: 745–751. 15130669 10.1016/j.exger.2004.02.011

[bib25] 25D'Antona G, Ragni M, Cardile A, Tedesco L, Dossena M, Bruttini F et al. Branched-chain amino acid supplementation promotes survival and supports cardiac and skeletal muscle mitochondrial biogenesis in middle-aged mice. Cell Metab 2010; 12: 362–372. 20889128 10.1016/j.cmet.2010.08.016

[bib26] 26Rennie MJ. Anabolic resistance: the effects of aging, sexual dimorphism, and immobilization on human muscle protein turnover. Appl Physiol Nutr Metab 2009; 34: 377–381. 19448702 10.1139/H09-012

[bib27] 27Phillips BE, Hill DS, Atherton PJ. Regulation of muscle protein synthesis in humans. Curr Opin Clin Nutr Metab Care 2012; 15: 58–63. 22037010 10.1097/MCO.0b013e32834d19bc

[bib28] 28Burd NA, Wall BT, van Loon LJ. The curious case of anabolic resistance: old wives' tales or new fables? J Appl Physiol (1985) 2012; 112: 1233–1235. 22134695 10.1152/japplphysiol.01343.2011

[bib29] 29Riazi R, Wykes LJ, Ball RO, Pencharz PB. The total branched-chain amino acid requirement in young healthy adult men determined by indicator amino acid oxidation by use of L-[1-13C]phenylalanine. J Nutr 2003; 133: 1383–1389. 12730426 10.1093/jn/133.5.1383

[bib30] 30Schauder P. Pharmacokinetic and metabolic interrelationships among branched-chain keto and amino acids in humans. J Lab Clin Med 1985; 106: 701–707. 4067381

[bib31] 31Glynn EL, Fry CS, Drummond MJ, Timmerman KL, Dhanani S, Volpi E et al. Excess leucine intake enhances muscle anabolic signaling but not net protein anabolism in young men and women. J Nutr 2010; 140: 1970–1976. 20844186 10.3945/jn.110.127647 PMC2955876

[bib32] 32Cynober L, Harris RA. Symposium on branched-chain amino acids: conference summary. J Nutr 2006; 136: 333S–336SS. 16365109 10.1093/jn/136.1.333S

[bib33] 33Dietary reference values for food energy and nutrients for the United Kingdom. Report of the Panel on Dietary Reference Values of the Committee on Medical Aspects of Food Policy. Rep Health Soc Subj (Lond) 1991; 41: 1–210. 1961974

[bib34] 34Hind K, Oldroyd B, Truscott JG. *In vivo* precision of the GE Lunar iDXA densitometer for the measurement of total body composition and fat distribution in adults. Eur J Clin Nutr 2011; 65: 140–142. 20842171 10.1038/ejcn.2010.190

[bib35] 35Rikli RE, Jones CJ. Senior Fitness Test Manual. 2nd edn, Human Kinetics: Champaign, IL, 2013, p 186.

[bib36] 36American College of Sports M, Roitman JL, Herridge M. ACSM's Resource Manual for Guidelines for Exercise Testing and Prescription, 4th edn. Lippincott Williams & Wilkins: Philadelphia, 2001, p 732.

[bib37] 37Borg GA. Psychophysical bases of perceived exertion. Med Sci Sports Exerc 1982; 14: 377–381. 7154893

[bib38] 38Hayden-Wade HA, Coleman KJ, Sallis JF, Armstrong C. Validation of the telephone and in-person interview versions of the 7-day PAR. Med Sci Sports Exerc 2003; 35: 801–809. 12750590 10.1249/01.MSS.0000064941.43869.4E

[bib39] 39Luiking YC, Deutz NE, Memelink RG, Verlaan S, Wolfe RR. Postprandial muscle protein synthesis is higher after a high whey protein, leucine-enriched supplement than after a dairy-like product in healthy older people: a randomized controlled trial. Nutr J 2014; 13: 9. 24450500 10.1186/1475-2891-13-9 PMC3909458

[bib40] 40Yang Y, Churchward-Venne TA, Burd NA, Breen L, Tarnopolsky MA, Phillips SM. Myofibrillar protein synthesis following ingestion of soy protein isolate at rest and after resistance exercise in elderly men. Nutr Metab (Lond) 2012; 9: 57. 22698458 10.1186/1743-7075-9-57 PMC3478988

[bib41] 41Bates B, Lennox A, Prentice A, Bates C, Swan G. National Diet and Nutrition Survey Headline Results from Years 1, 2, and 3 (combined) of the Rolling Programme (2008/2009-2010/11)2012 05-05-2014. Available from https://www.gov.uk/government/uploads/system/uploads/attachment_data/file/207708/NDNS-Y3-report_All-TEXT-docs-combined.pdf.

[bib42] 42EFSA. Scientific Opinion on Dietary Reference Values for protein EFSA Panel on Dietetic Products, Nutrition and Allergies. EFSA Journal [Internet]. 2012 05-05-2014; 10(2557): [66 p.]. Available from http://www.efsa.europa.eu/en/efsajournal/doc/2557.pdf.

[bib43] 43Halton TL, Hu FB. The effects of high protein diets on thermogenesis, satiety and weight loss: a critical review. J Am Coll Nutr 2004; 23: 373–385. 15466943 10.1080/07315724.2004.10719381

[bib44] 44Goodman CA, Frey JW, Mabrey DM, Jacobs BL, Lincoln HC, You JS et al. The role of skeletal muscle mTOR in the regulation of mechanical load-induced growth. J Physiol 2011; 589: 5485–5501. 21946849 10.1113/jphysiol.2011.218255 PMC3240886

[bib45] 45Biolo G, Maggi SP, Williams BD, Tipton KD, Wolfe RR. Increased rates of muscle protein turnover and amino acid transport after resistance exercise in humans. Am J Physiol 1995; 268: E514–E520. 7900797 10.1152/ajpendo.1995.268.3.E514

[bib46] 46O'Donovan G, Hillsdon M, Ukoumunne OC, Stamatakis E, Hamer M. Objectively measured physical activity, cardiorespiratory fitness and cardiometabolic risk factors in the Health Survey for England. Prev Med. 2013; 57: 201–205. 23732244 10.1016/j.ypmed.2013.05.022

[bib47] 47Bastone Ade C, Moreira Bde S, Vieira RA, Kirkwood RN, Dias JM, Dias RC. Validation of the human activity profile questionnaire as a measure of physical activity levels in older community-dwelling women. J Aging Phys Act 2014; 22: 348–356. 23917084 10.1123/japa.2012-0283

[bib48] 48Richardson MT, Ainsworth BE, Jacobs DR, Leon AS. Validation of the Stanford 7-day recall to assess habitual physical activity. Ann Epidemiol 2001; 11: 145–153. 11164131 10.1016/s1047-2797(00)00190-3

[bib49] 49Lang CH, Frost RA, Deshpande N, Kumar V, Vary TC, Jefferson LS et al. Alcohol impairs leucine-mediated phosphorylation of 4E-BP1, S6K1, eIF4G, and mTOR in skeletal muscle. Am J Physiol Endocrinol Metab 2003; 285: E1205–E1215. 12944322 10.1152/ajpendo.00177.2003

[bib50] 50Lang CH, Frost RA. Differential effect of sepsis on ability of leucine and IGF-I to stimulate muscle translation initiation. Am J Physiol Endocrinol Metab 2004; 287: E721–E730. 15186995 10.1152/ajpendo.00132.2004

[bib51] 51Pitkanen HT, Oja SS, Rusko H, Nummela A, Komi PV, Saransaari P et al. Leucine supplementation does not enhance acute strength or running performance but affects serum amino acid concentration. Amino Acids 2003; 25: 85–94. 12836063 10.1007/s00726-002-0343-3

[bib52] 52Alvestrand A, Hagenfeldt L, Merli M, Oureshi A, Eriksson LS. Influence of leucine infusion on intracellular amino acids in humans. Eur J Clin Invest 1990; 20: 293–298. 2114990 10.1111/j.1365-2362.1990.tb01858.x

[bib53] 53Nair KS, Matthews DE, Welle SL, Braiman T. Effect of leucine on amino acid and glucose metabolism in humans. Metabolism 1992; 41: 643–648. 1640850 10.1016/0026-0495(92)90057-h

[bib54] 54Dillon EL. Nutritionally essential amino acids and metabolic signaling in aging. Amino Acids 2013; 45: 431–441. 23239011 10.1007/s00726-012-1438-0 PMC3618985

